# Occurrence of *Staphylococcus* *aureus* on Farms with Small Scale Production of Raw Milk Cheeses in Poland

**DOI:** 10.3390/toxins8030062

**Published:** 2016-03-02

**Authors:** Jolanta G. Rola, Anna Czubkowska, Weronika Korpysa-Dzirba, Jacek Osek

**Affiliations:** Department of Hygiene of Food of Animal Origin, National Veterinary Research Institute, 24-100 Pulawy, Poland; anna.czubkowska@piwet.pulawy.pl (A.C.); weronika.korpysa@piwet.pulawy.pl (W.K.-D.); josek@piwet.pulawy.pl (J.O.)

**Keywords:** *S. aureus*, SE genes, antimicrobial resistance, raw milk cheese, public health

## Abstract

This paper describes the results of a 3-year study on the prevalence, enterotoxinogenicity and resistance to antimicrobials of *S. aureus* isolated on dairy farms with small scale production of raw cow milk cheeses. The samples of raw milk, semi-finished products and the final products as well as swabs were collected between 2011 and 2013 from nine dairy farms in Poland. A total of 244 samples were examined, of which 122 (50.0%) were contaminated with *S. aureus* including 18 of 26 (69.2%) mature cheese samples with log10 CFU g^−1^ between <1- and 7.41. In swabs collected from the staff and production environment the highest contamination rate with coagulase positive staphylococci (CPS) was detected on hands of cheese makers (4.34 log10 CFU/swab). None of the cheese samples contaminated with CPS contained staphylococcal enterotoxins (SEs). However, 55 of 122 (45.1%) *S. aureus* isolates possessed SEs genes, mainly (26 of 55; 47.3%) a combination of the *sed*, *sej* and *ser* genes. Furthermore, the *sep* (15 of 55; 27.3%) as well as *seg* and *sei* (9 of 55; 16.4%) genes were also identified. The remaining *S. aureus* isolates possessed the *sea* gene (one isolate), the combination of *sec*, *seg* and *sei* (three isolates) as well as the *sed*, *sej*, *sep* and *ser* markers together (one CPS). Resistance to penicillin (62 of 122 isolates; 50.8%) was the most common among the tested isolates. Some CPS were also resistant to chloramphenicol (7; 5.7%) and tetracycline (5; 4.1%). The obtained results indicated that the analyzed cheeses were safe for consumers. To improve the microbiological quality of traditional cheese products more attention should be paid to animal welfare and hygiene practices during the process of cheese manufacturing in some dairy farms.

## 1. Introduction

Staphylococcal food poisoning (SFP) is one of the most common foodborne diseases worldwide resulting from the consumption of foods containing staphylococcal enterotoxins (SEs) produced mainly by *Staphylococcus aureus* [1,2]. Symptoms of SFP have a rapid onset (2–8 h) and include hypersalivation, nausea, vomiting, abdominal cramping and diarrhea [3]. In the majority of cases recovery occurs within 24–48 h without specific treatment. Occasionally, the disease can be more severe or even fatal, especially in infants, elderly or immunecompromised patients. *S. aureus* is ubiquitous in the environment and can be found in the air, water, humans and animals. It is also one of the major causes of bovine mastitis and therefore, raw milk and subsequently raw milk products may be contaminated with *S. aureus* [4]. About 10% of cheeses in Europe are made from raw milk, which presents a considerable potential threat to human health [5]. In Scotland, *S. aureus* was found to be the most frequent pathogen of raw milk cheeses [6]. In France, a study of foodborne disease outbreaks showed that *S. aureus* was one of the most common causative pathogens associated with milk-related outbreaks [7]. The lack of proper hygienic measures during food processing may also increase the probability of contamination with *S. aureus*, especially in manually prepared foods. Cheese makers carrying enterotoxin-producing *S. aureus* in their noses or on their hands are thought to be the main source of food contamination caused byphysicial contact or through respiratory secretions. André *et al.* isolated *S. aureus* from hand and nose samples of approximately 75% of cheese makers in a dairy processing plant [8].

Some *S. aureus* produce toxins which are potent emetic agents causing SFP. In Italy, 55% of food isolates (milk, dairy products, meat, meat products) were positive for classic SEs (SEA-SEE) [9], while in Norway 48% of isolates from bovine raw milk and raw milk products were found to be SE producers [10]. Many *S. aureus* strains can synthesize more than one type of toxins [1,2] and to date 22 different SEs have been described. Apart from the classic SE types several new variants of SEs or staphylococcal-like toxins have been identified [11]. All of these toxins are heat stable and therefore, they may still be present in food while *S. aureus* is absent [3]. Enterotoxinogenic staphylococci need to reach levels of at least 5-6 log10 CFU g^−1^ to produce detectable amounts of SE [12]. More than 95% of SFP outbreaks worldwide are caused by classic enterotoxins, mainly SEA. On the other hand, strains of *S. aureus* isolated from cow milk are mostly positive for SEC and SED [12].

According to the recent European Food Safety Authority report, a total of 386 food-borne outbreaks caused by staphylococcal toxins were identified in the European Union in 2013 [13]. Most of them (336 outbreaks) were recorded in France where consumption of unpasteurized milk cheeses is common and milk-based products are more frequently involved in food poisoning than in other countries. In Poland, in the same year, only five confirmed food-borne intoxications were notified [13]. In recent years, traditionally made food products such as cheese manufactured at farm dairies are becoming more popular in our country [14]. These products should be of high quality and safe for consumers.

The aim of the study was to determine the occurrence of *S. aureus* in dairy farms with small scale production of raw cow milk cheese. Moreover, enterotoxinogenicity and resistance to antimicrobials of the isolates were investigated. 

## 2. Results

CPS were isolated from 122 (50.0%) out of 244 tested samples (Table 1). The bacteria were detected both in raw milk (12; 46.2%) and in the final cheese products (18; 69.2%). The highest percentage of CPS positive samples was found in the formed cheese (21 of 26; 80.8%), in grains after rinsing (19 of 25; 76.0%), milk curd (19 of 27; 70.4%) and in milk after heating (8 of 12; 66.7%). Of the 25 positive swabs CPS were most frequently isolated from the hands of cheese makers (11 of 26; 42.3%) and milk tanks (7 of 26; 26.9%) (Table 1).

Contamination with CPS depended on the type of material (Table 1). Only on two dairy farms the number of CPS in raw milk was 0 log10 CFU mL^−1^, whereas on other farms contamination was ranging from 0–1.0 to 0–5.0 log10 CFU mL^−1^. All semi-finished products obtained after addition of rennet were positive for CPS at the maximum levels of <1–5.83, 2.61–5.92 and 1.30–6.04 log10 CFU g^−1^ in milk curd, grains after rinsing and formed cheese, respectively. Furthermore, the final products in all dairy farms were contaminated with staphylococci and the levels ranged from 0 to <1–7.41 log10 CFU g^−1^. In swabs collected from production environment the highest contamination rate with CPS was detected on hands of cheese makers (4.34 log10 CFU/swab).

**Figure 1:**
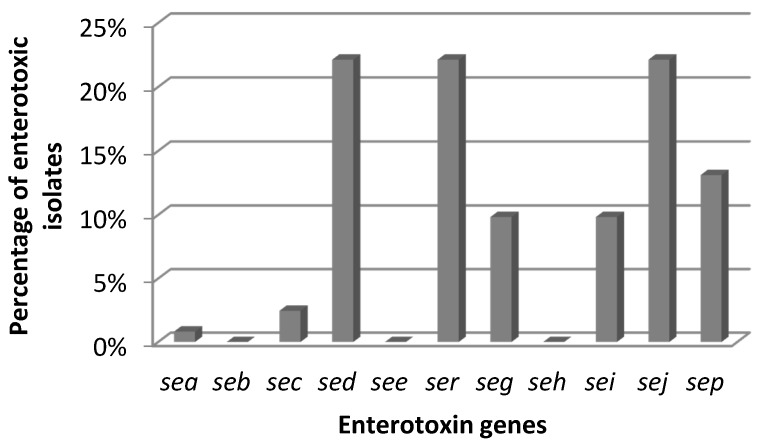
Enterotoxigenic *S. aureus* isolates.

All CPS were coagulase- and catalase-positive. β-haemolysis was observed in 108 of 122 (88.5%) isolates. Based on the presence of the *nuc* and *16S rRNA* genes all isolates were identified as *S. aureus*. Each sample contaminated with CPS, as well as all final products (total 105 samples) were tested for the presence of staphylococcal enterotoxins (SEs) and all of them were negative. However, SE genes were found in 55 (45.1%) isolates. Most of them were positive for three enterotoxin markers, *i.e.*, *sed* + *sej* + *ser* and *sec* + *seg* + *sei* (26 and three isolates, respectively). Some CPS had only one gene*—sep* (15; 12.0%) or *sea* (1; 1.8%) or four *sed* + *sej* + *sep* + *ser* toxin genes (1; 1.8%) (Figure 1). Detection of SEA-SEE in culture supernatants of the isolates possessing the *sea*—*see* genes resulted in the presence of enterotoxins in supernatants of 31 of 55 (56.4%) such isolates.

**Figure 2:**
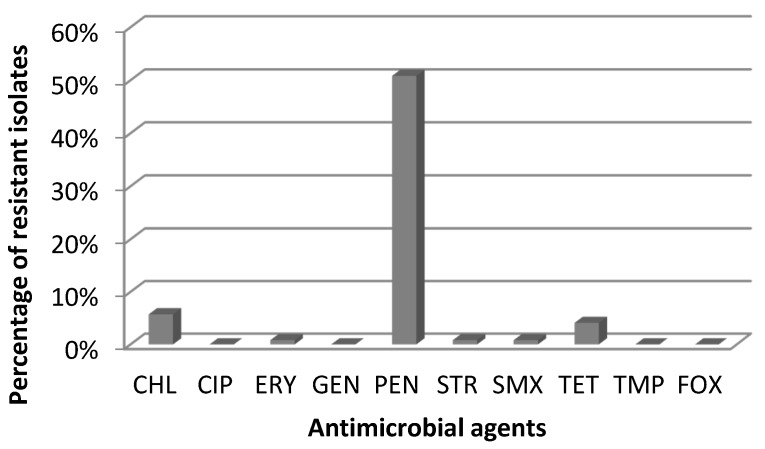
Antimicrobial resistance of *S. aureus* isolates.

Resistance to penicillin (62 of 122 isolates; 50.8%) was the most common among the tested *S. aureus*, followed by chloramphenicol (7; 5.7%), tetracycline (5; 4.1%), sulphamethoxazole (1; 0.8%) erythromycin (1; 0.8%) and streptomycin (1; 0.8%) (Figure 2). Of the 62 penicillin resistant *S. aureus* isolates, nine were also resistant to other antimicrobials: four to chloramphenicol, three to tetracycline, one to sulphamethoxazole and one to chloramphenicol together with streptomycin. 

## 3. Discussion

In this study, the prevalence and enterotoxigenicity of *S. aureus* isolated on dairy farms with small scale production of raw milk cheeses were tested. The results indicated that 46.2% of the raw milk samples were contaminated with CPS at the level up to 5 log10 CFU mL^−1^. It is interesting that in milk from two farms, CPS were not detected. A similar level of contamination of raw milk with CPS has been found by other authors [15,16,17,18,19]. However, there are also publications in which the rate of contamination was higher than in the present study. Peles *et al.* showed that bulk tank milk from 14 out of 20 farms was contaminated with *S. aureus* at levels up to 3.78 log10 CFU mL^−1^ [20]. Tondo *et al.* reported that *S. aureus* was present in 90.4% of raw milk samples with a mean number of 3.54 log10 CFU mL^−1^ [21]. These bacteria may be introduced to bulk milk either by direct excretion from the udder of a cow with clinical or subclinical staphylococcal mastitis or by fecal contamination [22]. In well drawn milk contamination with *S. aureus* ranged from 2.0 to 2.30 log10 CFU mL^−1^ but in the case of the presence of bacteria in the udder the number of these microorganisms may increase up to 4 log10 CFU mL^−1^ [23]. In the present study neither clinical nor subclinical mastitis were indicated in the cows from the dairy farms examined. Therefore, one of the possible explanations of the higher *S. aureus* level in some raw milk samples may be their contamination from the milking equipment or personnel involved in production.

The results of the current investigation showed that the contamination levels of the cheese gradually increased during its manufacture. It was observed that in farms in which both the quality of milk was high and the hands of cheese makers were negative for CPS, the number of staphylococci in mature cheese was lower or equal to 5 log10 CFU g^−1^. On the other hand, in farms with a high number of CPS both in milk and in hand’s swabs, the level of contamination increased along the production chain and peaked in the final products at as high as 6–7 log10 CFU g^−1^. An increase in contamination with *S. aureus*, of traditionally made cow milk cheese has been reported by others authors, especially at the stage from milk to curd [18]. These increasing levels may be explained by physical entrapment of *S. aureus* in the curd and the ability of these bacteria to grow rapidly in milk, as demonstrated by their generation time of 0.8 h at 25 °C [24,25]. Another possible source of contamination are people involved in cheese manufacturing, since *S. aureus* is frequently found on the skin of cheese makers [8,26]. In the present study, CPS were quite often detected on the hands of cheese makers in six out of nine tested farms and this could be the main source of contamination in the later stages of cheese manufacture.

None of the cheese contaminated with CPS contained staphylococcal enterotoxins but 45.1% isolated *S. aureus* harbored the SE genes. However, enterotoxins’ production by such strains is possible under appropriate environmental conditions, especially considering the temperature. The capability of the isolates for the production of enterotoxins A – E was confirmed in all *sea* – *see*-positive (31 of 122; 25.4%) isolates. Eight types of the SE genes (*sea*, *sec*, *sed*, *seg*, *sei*, *sej*, *sep*, *ser*) were detected in the isolated CPS and most of them (26 of 55; 47.3%) harbored the combination of the *sed*, *sej* and *ser* genes. The coexistence of the *sed* and *sej* markers has previously been reported by other authors and is due to the common location of the genes on the same plasmid [27]. The second largest subgroup of enterotoxigenic CPS detected in the present study was that with the *sep* gene (27.3%). Only one *S. aureus* strain had the *sea* gene. These results are similar to the study of Hummerjohann *et al.* who showed that *S. aureus* with the SE gene pattern *sed*, *sej* and *ser* dominated among the isolates recovered from Swiss raw milk cheeses [28]. Furthermore, several Italian studies also reported a dominance of the *sed* marker, which was often associated with the *sea* and *sej* genes [9,29,30]. In Norway, Jorgensen *et al.* indicated that 55% of CPS isolated from bovine bulk milk and 14.7% recovered from different stages of cheese production possessed SE genes but only the *seg* and *sei* markers were identified [19]. On the other hand, the present results are in contrast to the studies from Japan, Austria, France and Turkey, where the *sec* marker was commonly detected in *S. aureus* originating from milk and raw milk cheeses [10,31,32,33]. These data may possibly suggest a prevalence of certain *S. aureus* in different geographical regions.

The present study has shown that most *S. aureus* isolated from cheese were resistant to penicillin (50.8%); only a few of them showed resistance to tetracycline and sulfamethoxazole. Most of the CPS (47.5%) were resistant to one antimicrobial which is typical for strains of veterinary origin, whereas in human isolates rather multiresistant strains predominate [34]. Our results are comparable to those reported by other investigators [9,20]. The present results of antimicrobial resistance are in correlation with antibiotics that are used for treatment of bovine mastitis in Poland. Krasucka *et al.*, based on a questionnaire completed by 109 veterinarians from the whole of Poland, showed that penicillins are mostly used in treatment of cattle infections [35]. According to the report of the European Medicines Agency, in Poland in 2012 penicillins, tetracyclines and sulfonamides accounted for over 75% of the total of veterinary antimicrobial agents sold [36]. 

The obtained results have also indicated resistance to chloramphenicol in 5.7% of the isolates tested, although this antibiotic is banned in the European Union for animals bred for human consumption. However, this resistance may be the result of the presence of chloramphenicol naturally occurring in the environment [37].

The obtained results showed that the analyzed naturally ripened rennet cheese was safe for consumers. However, a significant proportion of samples was contaminated with *S. aureus* and therefore, more attention should be paid to animal welfare and hygiene of cheese manufacturing, especially quality of raw milk to improve the safety of these traditional products.

## 4. Materials and Methods

### 4.1. Sample Collection

A total of 244 samples from various stages of cheese production were collected between 2011 and 2013 from nine dairy farms located in the north-eastern part of Poland. They were small dairy farms rearing 4–6 milking cows and producing naturally ripened rennet cheese from raw unpasteurized cow milk. The production of such cheeses proceeds as follows: raw milk is heated to about 32 °C, the rennin added starts the process of cloth formation. Afterwards, the milk curds are cut and the liquid whey is separated. In the next stage, cheese grains are rinsed with warm water and once again liquid whey is separated. The grains are placed in special forms where the cheese is drained off, formed and the salt is added. After this step, the cheeses are transported to the specially assigned places for ripening. The following samples were collected from cheese production stages of which the highest level of *S. aureus* was expected: 26 of raw milk, 90 of semi-finished products (heated milk, curd, grains, formed cheese), 26 of final products and 102 of swabs from the production environment. Once on each farm, 8–10 samples were taken from the same stages of the production chain. Raw milk and semi-finished products were collected on the day of cheese production, whereas the final products were taken after two weeks of cheese ripening. The swabs were obtained using sterile 50 cm^2^ sampling sponges with maximum recovery diluent (TSC Ltd., Lancashire, UK). The swabs from the production environment, *i.e.*, milk tanks, were collected using 10 × 10 cm^2^ sterile propylene templates (TSC).

### 4.2. Enumeration of CPS

Enumeration of CPS was performed on Baird-Parker agar with rabbit plasma fibrinogen (bioMerieux, Marcy-I’Etoile, France). After 48 h of incubation at 37 ± 1 °C, one typical colony was taken for further identification [38].

### 4.3. Identification of S. aureus

*S. aureus* was identified by PCR detection of the *nuc* and *16S rRNA* genes according to the protocols recommended by the European Union Reference Laboratory for Antimicrobial Resistance [39].

### 4.4. Detection of Staphylococcal Enterotoxins Genes

The presence of SE genes was determined using two multiplex PCR assays. The first one was performed with six pairs of primers allowing detection of the genes: *sea*, *seb*, *sec*, *sed*, *see* and *ser* [40]. The second reaction enabled us to identify the *seg*, *seh*, *sei*, *sej* and *sep* genes [41].

### 4.5. Detection of Staphylococcal Enterotoxins A-E

The samples contaminated with CPS and all the final products were analyzed using a two-step method consisting of extraction/concentration and detection by enzyme linked fluorescent assay (ELFA) according to the European Union Reference Laboratory for Coagulase Positive Staphylococci protocol with the Vidas SET 2 test (bioMerieux) [42]. Detection of staphylococcal enterotoxins was also performed for *S. aureus* isolates harboring the sea-see genes after 72 h of incubation in brain heart infusion broth (BHI, Oxoid, Hampshire, UK) at 37 °C using the Ridasceen SET Total kit (R-Biopharm, Darmstadt, Germany).

### 4.6. Antimicrobial Resistance

A microbroth dilution method was used to establish the minimum inhibitory concentrations (MICs) of *S. aureus* isolates to 10 antimicrobial agents using the DKVP microplates (Trek Diagnostic Systems, Thermo Fisher Scientific, East Grinstead, UK). Antimicrobials, dilution ranges, and cut-off values used for MIC determination are described in Table 1. The isolates were cultured on Columbia agar supplemented with 5% sheep blood (bioMerieux) at 37 °C for 24 h. The MICs were established using Mueller-Hinton broth (Trek Diagnostic Systems, Thermo Fisher Scientific, East Grinstead, UK). The microplates were incubated at 36 °C for 18–20 h and read using the Vision^®^ system (Swin Version 3.3, Trek Diagnostic System, Thermo Fisher Scientific, East Grinstead, UK, 2011). The cut off values for the interpretation of the MIC results were in accordance with the European Committee on Antimicrobial Susceptibility Testing (EUCAST) and the EURL-AR (Table 2).

## Figures and Tables

**Table 1:** Contamination with coagulase positive staphylococci (CPS) at the different stages of cheese production.

Collected Material	Source	No. of Samples	No. (%) of CPS Positive Samples	Ranges of CPS at Different Production Stages								
				Farm No.								
				1	2	3	4	5	6	7	8	9
Swab				Log10 CFU/swab or CFU/cm^2^								
	Hands of cheese maker	26	11 (42.3)	0	0	0–4.34	0	0–3.23	0–2.72	0–2.70	0–3.81	0–3.12
	Milk tank	26	7 (26.9)	0	0	0.24–1.70	0	0–0.18	0	0–0.30	0–0.81	0–0.30
	Strainer/sacks	26	3 (11.5)	0	0	0–2.83	0	0	0–2.00	0	0	0
	Cheese mould	24	4 (16.7)	0–1.40	0	0–2.54	0	0–2.10	0	0	0	0–2.00
Sample				Log10 CFU mL^−1^ or CFU g^−1^								
	Raw milk	26	12 (46.2)	0	0	0–3.63	0–1.18	0–4.74	0–2.63	0	0–5.00	1.08–3.04
	Milk after heat treatment	12	8 (66.7)	0–1.70	0–0.18	0.70–1.48	1.18	2.36	1.73	0	0	3.90
	Curd	27	19 (70.4)	<1–2.64	2.18–2.53	<1–4.77	<1–1.60	<1–4.65	2.89–4.89	<1–3.83	<1–5.83	1–4.26
	Grains after rinsing	25	19 (76.0)	<1–3.36	<1–2.57	2.61–5.92	1.30–2.04	1.74–5.15	4.08–5.32	<1–4.74	<1	3.28–5.00
	Formed cheese	26	21 (80.8)	<1–3.95	<1–2.40	3.23–5.08	1–2.46	1.30–6.04	4.53–5.63	<1–4.74	<1–5.60	3.62–5.59
	Mature cheese	26	18 (69.2)	2.60–3.82	<1–5.53	1–3.08	<1	<1–4.91	<1–5.74	2.83–6.58	<1–7.41	4.81–7.11
Total		244	122 (50.0)									

**Table 2:** Antimicrobials, dilution ranges and cut-off values used for minimum inhibitory concentrations (MIC) determination of *S. aureus*.

Antimicrobial Class	Antimicrobials	Dilution Range (mg/L)	Cut off Values (mg/L) Resistant >
Amphenicols	Chloramphenicol (CHL)	2–64	16
Fluoroqinolones	Ciprofloxacin (CIP)	0.12–8	1
Macrolides	Erythromycin (ERY)	0.25–16	1
Aminoglycosides	Gentamicin (GEN)	0.25–16	2
	Streptomycin (STR)	4–64	16
β-Lactames	Penicillin (PEN)	0.06–16	0.125
Cephalosporins	Cefoxitin (FOX)	0.5–32	4
Tetracyclines	Tetracycline (TET)	0.5–32	1
Sulfonamides	Sulfamethoxazole (SMX)	32–512	128
Other	Trimethoprim (TMP)	0.5–32	4
